# Perovskite-type hydrides ACaH_3_ (A = Li, Na): computational investigation on materials properties for hydrogen storage applications†

**DOI:** 10.1039/d5ra01810b

**Published:** 2025-06-06

**Authors:** Sol-Hyang Ri, Un-Gi Jong, Thae-Song Im, Un-Ryong Rim

**Affiliations:** a Faculty of Distance Education, Kim Chaek University of Technology Pyongyang PO Box 76 Democratic People's Republic of Korea; b Faculty of Materials Science, Computational Materials Design (CMD), Kim Il Sung University Pyongyang PO Box 76 Democratic People's Republic of Korea ug.jong@ryongnamsan.edu.kp; c Faculty of Metal Engineering, Kim Chaek University of Technology Pyongyang PO Box 76 Democratic People's Republic of Korea; d Institute of Ocean Engineering, Kim Chaek University of Technology Pyongyang PO Box 76 Democratic People's Republic of Korea lur8971@star-co.net.kp

## Abstract

Recently, perovskite materials have emerged as a multifunctional material for photovoltaics, luminescence, photocatalytics and hydrogen storage applications. This work reports a theoretical investigation on materials properties of hydride perovskite ACaH_3_ (A = Li, Na) with cubic phase of *Pm*3̄*m* space group for application of H_2_ storage material. Electronic structure calculations show that the cubic LiCaH_3_ and NaCaH_3_ have an indirect bandgaps of 2.1 and 2.3 eV with valence band maximum at *R* point and conduction band minimum at *M* point. Based on geometric factors, elastic constants and self-consistent phonon calculations, we reveal that ACaH_3_ can be dynamically stabilized in cubic phase at elevated temperatures, and the compounds are mechanically stable as well, satisfying Born's stability criteria. Finally, our calculations demonstrate that gravimetric (volumetric) H_2_ storage capacities are 5.99 and 4.54 wt% (63.77 and 60.93 g L^−1^), and dehydrogenation temperatures are 453.76 and 688.16 K with a consideration of quantum effect for A = Li and Na, respectively. This work highlights that cubic LiCaH_3_ is regarded as a potential H_2_ storage material due to its high H_2_ storage capacity, stability and suitable dehydrogenation temperature.

## Abstract 2

We systematically investigated hydrogen storage properties of hydride perovskite ACaH_3_ (A = Li, Na), demonstrating materials stability, high storage capacities of 5.99 wt% and 63.77 g L^−1^ and suitable dehydrogenation temperature of 450 K for LiCaH_3_.

## Graphical abstract

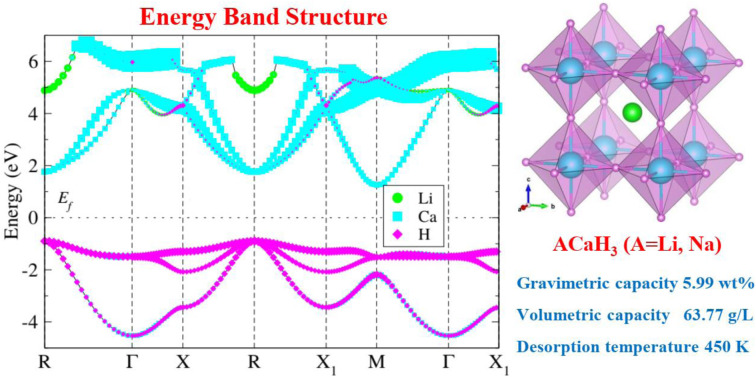

## 1 Introduction

Using hydrogen as a primary fuel in the long term would effectively reduce the world's dependence on fossil fuel without releasing any pollutant as by-products.^1–3^ In the quest for feasible hydrogen-fueled vehicles, one of the major challenges is to develop lightweight materials with high hydrogen densities (>5 wt%) which can absorb and release hydrogen in the range of 1–10 bar and 298–473 K.^4^ The U.S. Department of Energy (DOE) aims at developing a hydrogen storage material with a gravimetric capacity of 5.5 wt% and a volumetric capacity of 40 g L^−1^ by 2025 for a target driving range of 300 miles.^5^ Considerable research efforts have been concentrated on metal hydrides such as Mg(BH_4_)_2_,^6–9^ NaAlH_4_,^10–13^ LiBH_4_,^14–16^ MgH_2_ (ref. 17–19) and LiH_2_ (ref. 20) due to their relatively high hydrogen storage capacities and inexpensive production cost. However, most of these hydrides unfortunately suffer from poor kinetics and irreversibility of hydrogen absorption and desorption cycling. For example, it was demonstrated that LiBH_4_ (ref. 15) can be dehydrogenated and rehydrogenated above its melting point (∼550 K) because of its slow kinetics and unfavorable thermodynamics, and only when heating up to 770 K, most of the hydrogen stored in LiBH_4_ can be released from it. Moreover, some of them are likely to release toxic gas (*e.g.*, ammonia, diborane) during the dehydrogenation reaction.^21^ Nevertheless, the complex metal hydrides containing alkali metals are expected to have great potential for enhancing kinetics and reversibility as well as inhibiting release of pollutant.

In recent years, perovskite-type hydrides ABH_3_ where A and B are monovalent and divalent cations respectively have attracted great attention as a promising candidate for the future hydrogen storage materials.^22–29^ It was found that these compounds exhibit high thermodynamic stability, sufficient space to accommodate a large amount of hydrogen, catalytic effect on the hydrogen reversibility and high hydrogen storage capacity.^30–33^ Ikeda *et al.* first synthesized the perovskite-type hydride NaMgH_3_ by mechanically milling the binary hydrides of NaH and MgH_2_ at ambient temperature, explaining the formation ability of other hydride perovskite from Goldschmidt tolerance factors.^23–26^ They observed reversible hydrogenation and dehydrogenation with a hydrogen capacity of 5.9 wt% and rapid hydrogen motion at elevated temperature. On the other hand, Komiya *et al.* fabricated the perovskite-type hydrides AMgH_3_ (A = Na, K, Rb) by using ball-milling, reporting that the hydrides are dehydrogenated at temperatures between 673 and 723 K according to several ways depending on the type of A cation.^33^ Furthermore, it was revealed that the hydride perovskite ZrCoH_3_ can reversibly absorb and desorb hydrogen, but it suffers from the hydrogen-induced disproportionation (HID) phenomena.^34^ However, it was proved that the HID can be improved by partial substitution of Zr or Co cation with Ti cation.^35^

Trigger by the experimental studies,^23–26^ extensive theoretical investigations have been carried out to provide an in-depth understanding of materials properties for hydrogen storage applications of the perovskite-type hydrides.^22,28,30–32,36,37^ In 2007, Fornari *et al.*^31^ reported a density functional study on the structural and lattice dynamics properties for the perovskite hydrides AMgH_3_ (A = Na, K, Rb), revealing the compound's ionic bonding nature and dynamical stability. Based on density functional theory (DFT) calculations, Gencer *et al.*^38^ investigated the electronic and mechanical properties of the hydride perovskites LiNiH_3_, NaNiH_3_ and KNiH_3_ with hydrogen storage capacities of 4.4, 3.6 and 3.3 wt% respectively, illustrating their mechanical stability and metallic nature. Siddique *et al.*^39,40^ carried out DFT studies on the dynamical and mechanical stabilities of the hydride perovskites LiBH_3_ (B = Sc, Ti, V) and AVH_3_ (A = Be, Mg, Ca, Sr) with hydrogen storage capacities higher than 4.0 wt%, confirming their stabilities. Recently, Xu *et al.*^36^ reported the hydrogen storage capacities and structural, electronic, optical, mechanical, thermodynamic and dynamical properties for XAlH_3_ (X = Na, K) hydride perovskites. They concluded that the compounds show mechanical, dynamical and thermodynamical stabilities with metal-like electronic properties by ionic chemical bonding. Despite enormous researches on the hydride perovskites, little attention has been drawn to perovskite-type hydride ACaH_3_ (A = Li, Na) for application as a hydrogen storage material. The LiCaH_3_ and NaCaH_3_ have high hydrogen storage capacities of 6.0 and 4.5 wt%, respectively and contain nontoxic and earth-abundant elements. Thus, the hydride perovskites ACaH_3_ (A = Li, Na) can be regarded as a potential candidate for high-performance, low-cost and nontoxic hydrogen storage materials. In this work, we present a comprehensive and systematic investigation of the structural, electronic and lattice dynamic properties and thermodynamic and mechanical stabilities of the hydride perovskites ACaH_3_ for the application as the hydrogen storage material by using first-principles calculations.

## 2 Computational methods

Density functional theory (DFT) calculations were performed using the Vienna *ab initio* simulation package (VASP).^44,45^ The projector augmented wave (PAW) potentials^46,47^ were used to describe the interactions between ions and valence electrons. The valence electron configurations were given as Li–2s^1^, Na–3s^1^, Ca–3p^6^4s^2^ and H–1s^1^. Convergence test revealed that the cutoff energy 800 eV for plane wave basis sets and *k*-point mesh 10 × 10 × 10 provided a total energy accuracy of 1 meV per atom (see Fig. S1, ESI†). The variable-cell structural optimizations were performed until the atomic forces were less than 10^−3^ eV Å^−1^ with the self-consistent convergence threshold of 10^−8^ eV. We utilized the Perdew–Burke–Ernerhof (PBE)^48^ and PBE-revised functionals for solid (PBEsol)^49^ within the generalized gradient approximation (GGA) and the Perdew–Wang (PW91)^50^ functional within the local density approximation (LDA) in order to account for the exchange-correlation (XC) interaction among the valence electrons. We computed the atomic forces for the 2 × 2 × 2 supercells, using the reduced cutoff energy of 600 eV and *k*-point mesh of 4 × 4 × 4 with the same convergence thresholds.

As proposed by Ikeda,^23^ we considered the H_2_ decomposition reaction for the hydride perovskite ACaH_3_ as follows:1
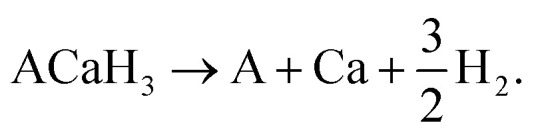


According to eqn (1), the H_2_ decomposition enthalpy Δ*H* was calculated as follows:2
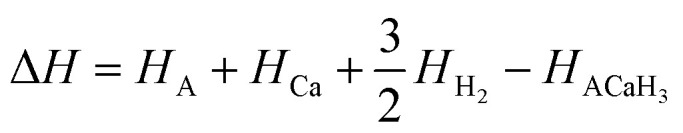
where *H*_material_ is an enthalpy for the corresponding material. Materials enthalpy *H* was computed as follows:

3 *H* = *E*_textele_ + *E*_zpe_

where *E*_ele_ and *E*_zpe_ are the electronic total energy and the zero-point energy by quantum effect, respectively. Then, we estimated the *E*_zpe_ by using the following formula:4
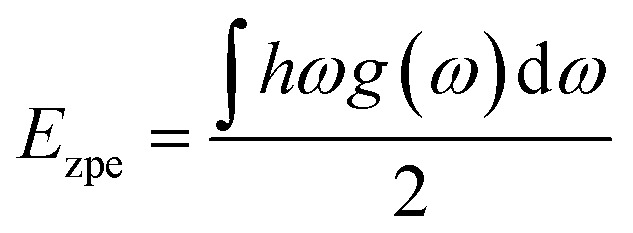
where *h*, *ω* and *g*(*ω*) are the Plank's quantum constant, phonon frequency and phonon density of state, respectively. The H_2_ decomposition temperature *T*_des_ can be estimated as follows:

5 *T*_des_ = −Δ*H*/Δ*S*

where Δ*H* is the H_2_ decomposition enthalpy and Δ*S* is the entropy increment in the decomposition reaction. In this work, we approximate the Δ*S* to the entropy of H_2_ gas, 130.7 J mol^−1^ K^−1^.^51^

Hydrogen storage materials are utilized to store and release hydrogen for the applications of the hydrogen-powered vehicles, fuel cells and energy storage medium. Thus, their mechanical stabilities are a crucial factor because they determine the hydrogen storage system's durability and safety under operation. In order to estimate the materials mechanical stability, we evaluated the elastic constants such as bulk (*B*), shear (*G*) and Young's (*E*) moduli, which represent linear response of lattice to small strain. The elastic stiffness (*C*_*ij*_) and compliance (*S*_*ij*_) constants were obtained from the density functional perturbation theory (DFPT)^52^ calculations using the PBEsol functional.6
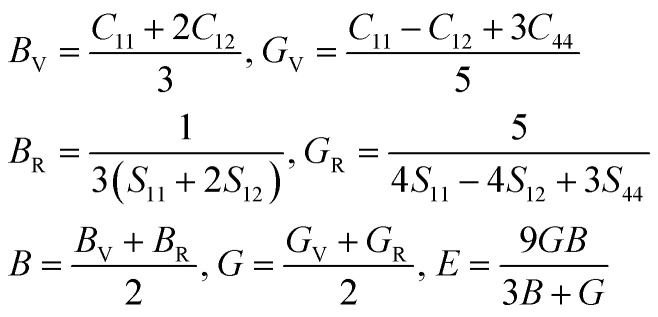


For the cubic phase, there exist three independent stiffness constants such as *C*_11_, *C*_12_ and *C*_44_. According to the Born's stability criteria,^53^ the cubic crystalline solids are mechanically stable when satisfying the following eqn (7):

7 *C*_11_ > 0, *C*_44_ > 0, *C*_11_ + 2*C*_12_ > 0, *C*_11_ − *C*_12_ > 0

In order to calculate the phonon density of state (DOS) and the *E*_zpe_, we computed the phonon dispersion curves for all the compounds involved in the H_2_ decomposition reaction eqn (1). The phonon dispersion curves and DOS calculations were carried out by using the finite displacement method, as implemented in the ALAMODE code.^54,55^ Using 2 × 2 × 2 supercells, we prepared 30 different configurations where all the atoms were randomly displaced by 0.01–0.06 Å from their equilibrium positions for the cubic ACaH_3_, A and Ca compounds, respectively. We then calculated the atomic forces for all the displaced configurations by performing precise DFT calculations, and harmonic and anharmonic interatomic force constants (IFCs) were extracted by using the compressive sensing lattice dynamics (CSLD),^56^ as implemented in the ALAMODE code. We ensure that for all the compounds, the IFCs can reproduce the atomic forces within the relative errors less than 2.4% when comparing to the DFT-calculated forces. The harmonic IFCs were extracted by considering all the possible harmonic terms, whereas the cubic and quartic anharmonic IFCs were extracted by setting the cutoff distance up to the 8th – and 5th – nearest neighbor for each type of atom. The self-consistent phonon (SCP) calculations^57^ were carried out to obtain temperature-dependent phonon dispersion curves and DOS by considering the anharmonic effects at finite temperatures.

## 3 Results and discussion

### 3.1 Crystal structures

**Fig. 1:**
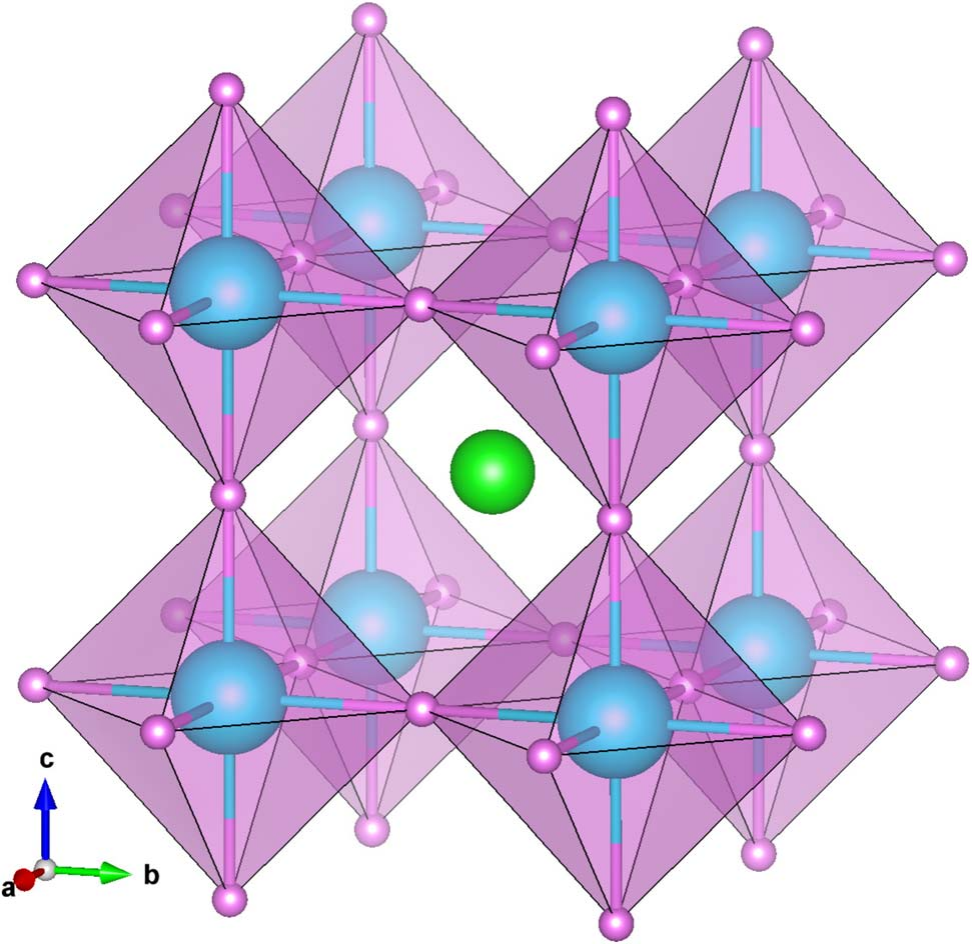
Polyhedral view of crystalline structure optimized using PBEsol functional for cubic ACaH_3_ with a space group of *Pm*3̄*m*. The green, cyan and purple balls represent the A, Ca and H atoms, respectively.

Perovskite-type compounds are generally regarded to successively crystallize in orthorhombic, tetragonal and cubic phases upon increasing temperature. In fact, it was experimentally found that MMgH_3_ (M = Na, K, Rb) adopt the cubic and orthorhombic phases at different temperatures.^21,23,33^ As for the perovskite compounds ABH_3_, the stability of the perovskite structure can be empirically assessed by using the Goldschmidt tolerance factor 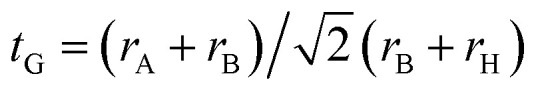 and octahedral factor *t*_o_ = *r*_B_/*r*_H_, where *r*_A_, *r*_B_ and *r*_H_ are the ionic radii for A^+^, B^2+^ and H^−^ ions, respectively. The *t*_o_ can be used to assess whether the BH_6_ octahedron is stable, while the *t*_G_ to check whether the A-site cation can fit between the BH_6_ octahedra. According to the empirical criteria,^58,59^ the perovskite compounds can adopt the stable cubic phase when satisfying 1.0 ≥ *t*_G_ ≥ 0.8 and *t*_o_ ≥ 0.4. This criteria have been successfully applied to estimate the formability of cubic phase for the oxide, halide and fluoride perovskites.^60,61^Table 1 lists the calculated geometric factors of *t*_G_ and *t*_o_ for the hydride perovskites ACaH_3_ (A = Li, Na). Based on the analysis of geometric factors, it was found that the hydride perovskites LiCaH_3_ and NaCaH_3_ can stabilize in the cubic phase because of suitable geometric factors of *t*_G_ = 0.8, 0.9 and *t*_o_ = 0.7. Therefore, it was supposed that the hydride perovskites ACaH_3_ can adopt the cubic phase with the *Pm*3̄*m* space group like the oxide, halide and fluoride perovskites (see Fig. 1). We note that even though the structural factors of *t*_G_ and *t*_o_ can provide a qualitative estimation of the perovskite structure stability, a quantitative and detailed assessment of materials stability should be based on precise calculations of lattice dynamics properties and elastic constants.

**Table 1:** Structural parameters calculated using the PW91, PBE and PBEsol functionals and geometric factor *t*_textG_ and *t*_texto_ for the cubic LiCaH_3_, NaCaH_3_, Li, Na and Ca compounds with previous experimental data.^3^

Compound	PW91					PBE				PBEsol				Exp.				Geometric factor	
	Lattice constants (Å)	Atom	Position			Lattice constants (Å)	Position			Lattice constants (Å)	Position			Lattice constants (Å)	Position				
			*x*	*y*	*z*		*x*	*y*	*z*		*x*	*y*	*z*		*x*	*y*	*z*	*t*_G_	*t*_o_
LiCaH_3_	*a* = 4.25	Li	0.5	0.5	0.5	*a* = 4.29	0.5	0.5	0.5	*a* = 4.28	0.5	0.5	0.5	—	—	—	—	0.8	0.7
		Ca	0.0	0.0	0.0		0.0	0.0	0.0		0.0	0.0	0.0	—	—	—	—		
		H1	0.5	0.0	0.0		0.5	0.0	0.0		0.5	0.0	0.0	—	—	—	—		
		H2	0.0	0.5	0.0		0.0	0.5	0.0		0.0	0.5	0.0	—	—	—	—		
		H3	0.0	0.0	0.5		0.0	0.0	0.5		0.0	0.0	0.5	—	—	—	—		
NaCaH_3_	*a* = 4.31	Na	0.5	0.5	0.5	*a* = 4.35	0.5	0.5	0.5	*a* = 4.34	0.5	0.5	0.5	—	—	—	—	0.9	0.7
		Ca	0.0	0.0	0.0		0.0	0.0	0.0		0.0	0.0	0.0	—	—	—	—		
		H1	0.5	0.0	0.0		0.5	0.0	0.0		0.5	0.0	0.0	—	—	—	—		
		H2	0.0	0.5	0.0		0.0	0.5	0.0		0.0	0.5	0.0	—	—	—	—		
		H3	0.0	0.0	0.5		0.0	0.0	0.5		0.0	0.0	0.5	—	—	—	—		
Li	*a* = 3.44	Li1	0.5	0.5	0.5	*a* = 3.49	0.5	0.5	0.5	*a* = 3.47	0.5	0.5	0.5	*a* = 3.47^a^	0.5	0.5	0.5		
		Li2	0.0	0.0	0.0		0.0	0.0	0.0		0.0	0.0	0.0		0.0	0.0	0.0		
Na	*a* = 4.17	Na1	0.5	0.5	0.5	*a* = 4.25	0.5	0.5	0.5	*a* = 4.23	0.5	0.5	0.5	*a* = 4.22^b^	0.5	0.5	0.5		
		Na2	0.0	0.0	0.0		0.0	0.0	0.0		0.0	0.0	0.0		0.0	0.0	0.0		
Ca	*a* = 5.51	Ca1	0.0	0.0	0.0	*a* = 5.61	0.0	0.0	0.0	*a* = 5.59	0.0	0.0	0.0	*a* = 5.57^c^	0.0	0.0	0.0		
		Ca2	0.5	0.5	0.0		0.5	0.5	0.0		0.5	0.5	0.0		0.5	0.5	0.0		
		Ca3	0.5	0.0	0.5		0.5	0.0	0.5		0.5	0.0	0.5		0.5	0.0	0.5		
		Ca4	0.0	0.5	0.5		0.0	0.5	0.5		0.0	0.5	0.5		0.0	0.5	0.5		

a Experiment.^41^

b Experiment.^42^

c Experiment.^43^

Through the variable-cell structural optimization by using the PW91, PBE and PBEsol functionals, we calculated the equilibrium lattice constants for the cubic ACaH_3_, Ca, Li and Na compounds. According to the previous experiments,^41–43^ we assume that the Ca and Li (Na) compounds adopt the cubic phases with the space group of *Fm*3̄*m* and *Im*3̄*m*, respectively (see Fig. S2, ESI†). The calculated lattice constants are listed in Table 1. The PBEsol-calculated lattice constant of LiCaH_3_ is *a* = 4.28 Å, which is slightly smaller than *a* = 4.34 Å of NaCaH_3_ because of the smaller ionic radius of Li cation than Na cation. To the best of our knowledge, there are no experimental and theoretical works of the hydride perovskites ACaH_3_ for comparison. However, the optimized lattice constants are in good agreement with the previous experiments^41–43^ for the cubic Ca, Li and Na. Especially, the PW91-calculated lattice constants of *a* = 5.51, 4.17 and 3.44 Å underestimate the experimental ones^41–43^ of *a* = 5.59, 4.23 and 3.47 Å for the cubic Ca, Na and Li respectively, whereas the PBE-calculated ones slightly overestimate the experimental ones. Moreover, the PBEsol-calculated lattice constants of *a* = 5.59, 4.23 and 3.47 Å are in excellent agreement with the experimental ones of *a* = 5.59, 4.23 and 3.47 Å, providing a relative error less than 0.5%. Hereby, we adopted the PBEsol functional for the calculations of lattice dynamics and electronic structure properties, mechanical stability and H_2_ decomposition energetics for the cubic ACaH_3_.

### 3.2 Lattice dynamics properties

**Fig. 2:**
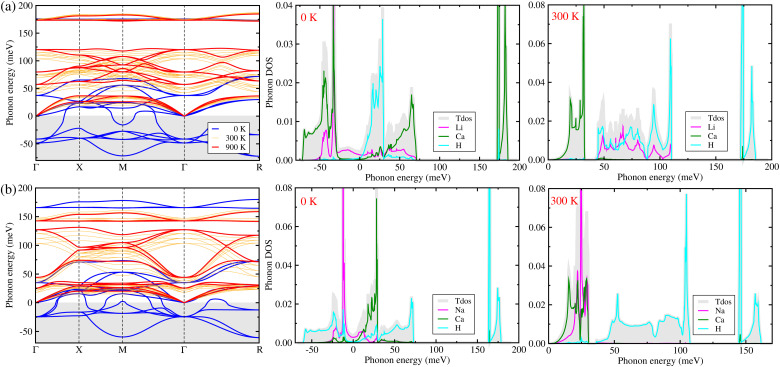
Phonon dispersion curves and atom-projected phonon density of states (DOS) calculated by using the self-consistent phonon (SCP) theory at elevated temperatures for the hydride perovskites (a) LiCaH_3_ and (b) NaCaH_3_ with the cubic phase of a space group of *Pm*3̄*m*. The thick blue and red lines display the phonon dispersion curves calculated at 0 and 900 K, respectively, while the thin orange lines at 300, 500 and 700 K.

**Fig. 3:**
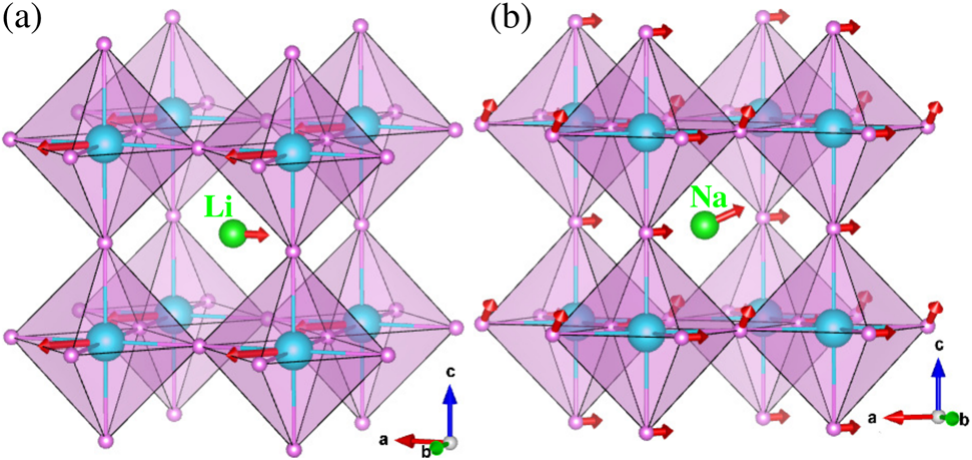
Polyhedral view of strong anharmonic vibrations at 0 K associated with the lowest soft phonon modes at the *Γ* point for the cubic (a) LiCaH_3_ and (b) NaCaH_3_. The red arrows display the atomic displacement vectors. The green, cyan and purple balls represent the Li (Na), Ca and H atoms, respectively.

In order to predict lattice dynamics properties, we calculated the phonon dispersion curves and density of states (DOS) at elevated temperatures for the cubic ACaH_3_ (A = Li, Na) with a space group of *Pm*3̄*m* by using the SCP theory. From the phonon dispersions and DOS (Fig. 2), we can directly estimate the material's dynamical stability and compute the zero-point *E*_zpe_ energy in consideration of quantum effect by using eqn (4). As calculated at 0 K, Fig. 2 shows the harmonic phonon dispersion curves (blue-colored lines in the left panel) computed along the high symmetry line of *Γ*–*X*–*M*–*Γ*–*R* in the phonon Brillouin zone (BZ). It was found that the relatively deep negative phonon eigenvalues reaching up to −70 meV, known as the soft mode, appear in the whole phonon BZ range for the cubic ACaH_3_. Such finding indicates that these compounds are dynamically unstable in the cubic phase at 0 K. From the phonon DOS at 0 K (middle panel in Fig. 2), we found that these soft phonon modes are mainly ascribed to the Ca- and Li-atomic (Na- and H-atomic) vibrations for the LiCaH_3_ (NaCaH_3_).The symmetry analysis of the phonon eigenvectors indicates that the soft phonon modes are responsible for symmetry breaking instabilities like in the halide and oxide perovskites.^62,63^ As shown in Fig. 3(a), the lowest soft mode at the *Γ* point induces the displacement of Li and Ca atoms from the ideal centers of the CaH_3_ inorganic framework and the CaH_6_ octahedron, respectively for the cubic LiCaH_3_. Moreover, the lowest soft modes causes collective displacements of the H and Na atoms with a larger magnitude of Na displacement for the cubic NaCaH_3_ (Fig. 3(b)). The presence of such soft modes suggests that the cubic hydride perovskites ACaH_3_ can undergo a phase transition to a lower symmetry structure such as tetragonal or orthorhombic phase upon decreasing temperature like the halide and oxide perovskites. The calculated phonon dispersion curves and DOS are plotted in Fig. S3–S5, ESI† for the cubic Li, Na and Ca without any soft phonon modes, implying their dynamical stabilities.

To clearly estimate the phase stability, we renormalized the phonon dispersion curves and DOS at elevated temperatures from 300 to 900 K with an interval of 200 K by using the SCP theory. From the SCP calculations, the negative phonon energies corresponding to the soft modes were renormalized to be real within the whole range of the phonon BZ (orange and red lines in the left panel of Fig. 2), which indicates that the cubic phase of ACaH_3_ is dynamically stable at elevated temperatures, as confirmed in experiment^33^ for the Mg-based hydride perovskites AMgH_3_ (A = Na, K, Rb). In particular, the negative phonon energies of −49 and −38 (−26) meV were renormalized at 900 K to 81 and 129 (40 and 125) meV at the *Γ* point for LiCaH_3_ (NaCaH_3_). Noticeable changes were observed for the phonon DOS as well (right panel in Fig. 2). For instance, at 300 K, the H and Ca atoms in both LiCaH_3_ and NaCaH_3_ play dominant roles in the high-lying optical phonon modes and the acoustic phonon modes coupled with the low-lying optical modes, respectively. However, in the case of LiCaH_3_, the Li atom makes a dominant contribution to the mid-lying phonon modes ranging from 40 to 115 meV, while in the case of NaCaH_3_, the Na atom significantly contributes to the acoustic and low-lying optical modes below 30 meV. Upon increasing temperature, the phonon dispersion curves and phonon DOS broaden towards higher energy due to stronger atomic vibrations (see Fig. S6 and S7, ESI†).

### 3.3 Electronic structure properties

**Fig. 4:**
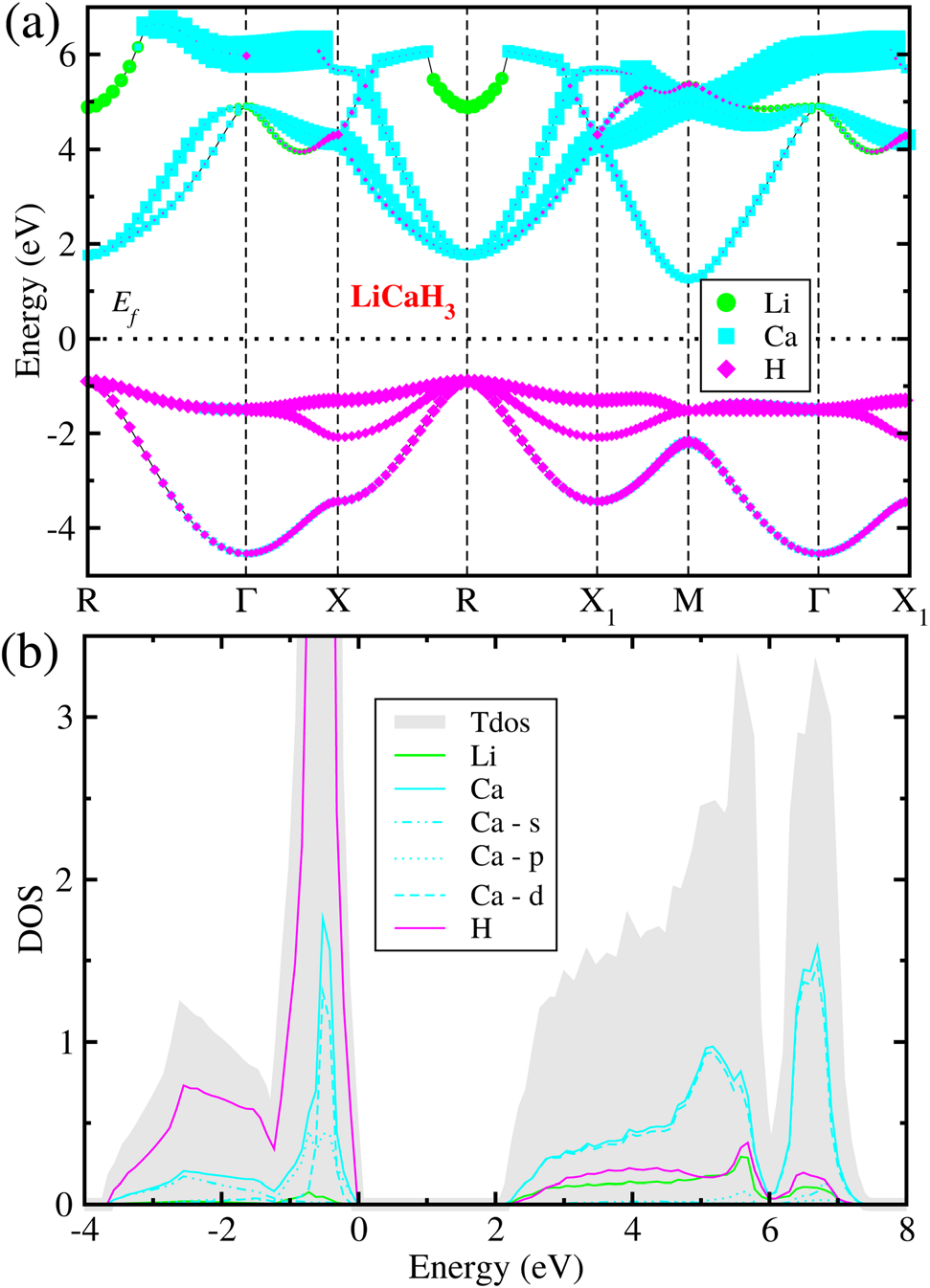
(a) Atom-resolved electronic band structure and (b) partial density of states (DOS) calculated with the PBEsol functional for the hydride perovskite LiCaH_3_ in the cubic phase.

**Fig. 5:**
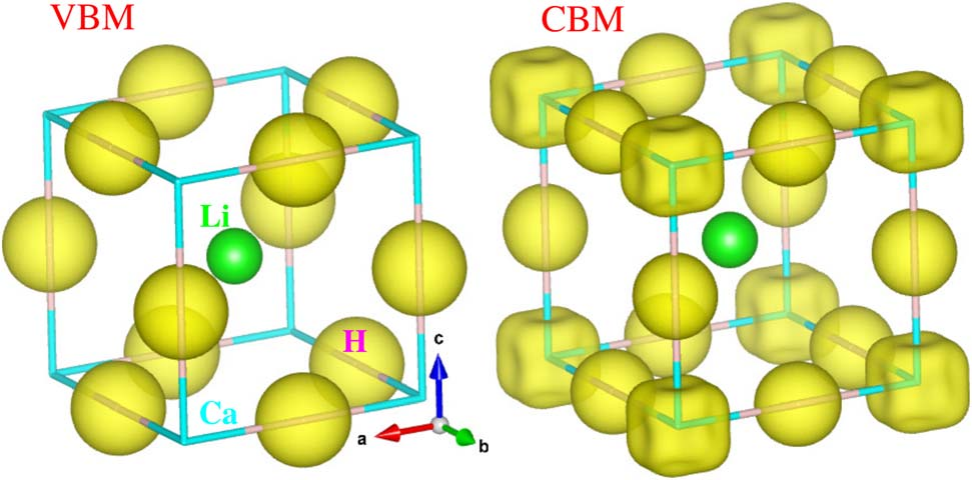
Isosurface plot of charge density corresponding to the conduction band minimum (CBM) and valence band maximum (VBM) at the values of 0.02 |*e*| Å^−3^ in the cubic ACaH_3_. The green, cyan and purple balls represent the Li (Na), Ca and H atoms, respectively.

Considering that the cubic phase can be stabilized at elevated temperatures, we calculated the electronic structure properties for the cubic hydride perovskite ACaH_3_ with the space group of *Pm*3̄*m* by using the PBEsol functional. Using the calculated energy band structures (Fig. 4(a)), we can directly estimate the energy band gap, and from the atom-projected electron DOS (Fig. 4(b)), we can understand which atom can play important role near the Fermi level *E*_f_. It was established that the PBE or PBEsol functional within the GGA can describe electronic structure properties in good agreement with experiment for the halide and hydride perovskites. The spin–orbit coupling (SOC) effect was not considered as all the constituent atoms are very light for ACaH_3_. Fig. 4(a) shows the energy band structure calculated along the high symmetry line of *R*–*Γ*–*X*–*R*–*X*_1_–*M*–*Γ*–*X*_1_ in the BZ for the cubic LiCaH_3_. It was found that the compound has an indirect band gap of 2.1 eV with the valence band maximum (VBM) at the *R* point and the conduction band minimum (CBM) at the *M* point, and a direct band gap of 2.8 eV at the *R* point. Meanwhile, the cubic NaCaH_3_ provides an indirect (direct) band gap of 2.3 (3.2) eV with similar band structures to the LiCaH_3_ (see Fig. S8, ESI†). It is worth noting that the larger the ionic radii of A cation, the larger band gaps the hydride perovskites ACaH_3_ provide, being similar to the case of the halide and fluoride perovskites.^60,61^ Through the analysis of atom-projected partial DOS, it was revealed that the valence bands (VBs) are dominated by H-s state with a small contribution of Ca-s states, while the conduction bands (CBs) are dominated by Ca-s states coupled with Li- and H-s states (see Fig. 4 and S8, ESI†).Such analysis coincides with the isosurface plot of the electron charge density corresponding to the VBM and CBM, as shown in Fig. 5.

### 3.4 Mechanical stability and H_2_ decomposition energetics

In order to assess the mechanical stability of the compounds, we then estimated the elastic constants such as the stiffness constant (*C*_*ij*_), bulk modulus (*B*), shear modulus (*G*) and Young's modulus (*E*) by using the DFPT calculations. As listed in Table 2, the elastic stiffness constants of *C*_11_, *C*_12_ and *C*_44_ satisfy the Born's stability criteria for the cubic LiCaH_3_ and NaCaH_3_, expressed as eqn (7). Furthermore, we found that the cubic LiCaH_3_ (NaCaH_3_) is a ductile (brittle) material according to the Pugh's criteria^64^ because the Poisson's ratio *ν* and the Pugh's ratio *B*/*G* are larger (smaller) than the threshold values of 0.26 and 1.75, respectively (see Table 2). Based on such analysis, it was found that the cubic ACaH_3_ is mechanically as well as dynamically stable. Due to the larger *B* (smaller *G* and *E*), the LiCaH_3_ exhibits greater (smaller) hardness upon bulk (shear and tensile) deformation compared to the NaCaH_3_. On the other hand, both LiCaH_3_ and NaCaH_3_ are elastically anisotropic in nature because the anisotropic factor *A* = 2*C*_44_/(*C*_11_ − *C*_12_) is much smaller than unity.

**Table 2:** Gravimetric and volumetric H_2_ storage density (*ρ*_g_ and *ρ*_v_), elastic stiffness constant (*C*_*ij*_), bulk modulus (*B*), shear modulus (*G*), Young's modulus (*E*), Pugh's ratio (*B*/*G*), Poisson's ratio (*ν*), zero-point energy (*E*_zpe_), H_2_ decomposition enthalpy (Δ*H*) and decomposition temperatures (*T*_des_ and *T*^q^_des_ without and with quantum effect) for the cubic ACaH_3_

Functional	Properties	LiCaH_3_	NaCaH_3_	Li	Na	Ca	H_2_
	*ρ*_g_ (wt%)	5.99	4.54				
	*ρ*_v_ (g L^−1^)	63.77	60.93				
PW91	*H* (eV)	−14.92	−14.85	−3.77	−2.61	−7.73	−6.81
	Δ*H* (eV)	0.89	1.39				
	*T*_des_ (K)	437.42	687.23				
PBE	*H* (eV)	−14.73	−14.64	−3.81	−2.62	−7.73	−6.77
	Δ*H* (eV)	0.73	1.24				
	*T*_des_ (K)	361.68	612.46				
PBEsol	*H* (eV)	−14.74	−14.66	−3.92	−2.80	−8.54	−6.51
	Δ*H* (eV)	0.87	1.35				
	*T*_des_ (K)	430.86	666.23				
	*E*_zpe_ (eV)	0.37	0.37	0.34	0.02	0.01	0.27
	*T* ^q^_des_ (K)	453.76	688.16				
	*C*_11_ (GPa)	661.99	631.11				
	*C*_12_ (GPa)	83.89	95.43				
	*C*_44_ (GPa)	90.46	122.79				
	*B* (GPa)	276.59	273.99				
	*G* (GPa)	147.32	168.78				
	*E* (GPa)	375.33	420.09				
	*B*/*G*	1.87	1.62				
	*ν*	0.27	0.24				

Finally, we investigated the H_2_ storage capacities and decomposition energetics for the hydride perovskites ACaH_3_ (A = Li, Na). The H_2_ gravimetric and volumetric storage capacities were calculated by using the formula of *ρ*_g_ = 3*M*_H_/(*M*_A_ + *M*_Ca_ + 3*M*_H_) × 100% and *ρ*_v_ = 3*M*_H_/(*N*_A_*V*_o_), where *M*_A_, *M*_Ca_ and *M*_H_ are the molar masses of A, Ca and H atoms, respectively while *N*_A_ and *V*_o_ are the Avogadro number and PBEsol-optimized unit cell volume. As shown in Table 2, the LiCaH_3_ and NaCaH_3_ have *ρ*_g_ (*ρ*_v_) of 5.99 and 4.54 wt% (63.77 and 60.93 g L^−1^), respectively, being larger or comparable to the targeted values of 5.5 wt% (40 g L^−1^) provided by the U.S. DOE.

The H_2_ decomposition temperature *T*^q^_des_ (*T*_des_) with (without) a consideration of the quantum effect was estimated by employing the eqn (5). Ignoring the quantum effect, the PBEsol-calculated *T*_des_ is 430.86 and 666.23 K for the cubic LiCaH_3_ and NaCaH_3_, respectively. With the calculated phonon energies and DOS, we calculated the zero-point energy *E*_zpe_, finding that by considering the quantum effect, the *T*^q^_des_ was slightly increased to 453.76 and 688.16 K for LiCaH_3_ and NaCaH_3_, respectively. To sum up, the hydride perovskite LiCaH_3_ can store 5.99 wt% and 63.77 g L^−1^ hydrogen with mechanical and dynamical stabilities and suitable H_2_ decomposition temperature of about 450 K, satisfying the U.S. DOE requirement.

## 4 Conclusions

In conclusion, by using the first-principles calculations, we have theoretically investigated the materials properties such as structural, electronic and lattice dynamics properties and mechanical and dynamical stabilities of the hydride perovskites ACaH_3_ (A = Li, Na) in cubic phase of *Pm*3̄*m* space group for the application of hydrogen storage material. From the calculations of Goldschmidt tolerance factor *t*_G_ and octahedral factor *t*_o_, it was suggested that the ACaH_3_ (A = Li, Na) can stabilize in the cubic phase of *Pm*3̄*m* space group. We found that the PBEsol-calculated lattice constants and atomic positions are in good accordance with the available experimental data, while the PBE(PW91) functional slightly overestimate (underestimate) the structural properties for Li, Na and Ca metals. From the electronic structure calculations, it was found that the cubic LiCaH_3_ and NaCaH_3_ have an indirect (direct) bandgaps of 2.1 and 2.3 (2.8 and 3.2) eV with the VBM at the *R* point and the CBM at the *M* point (at the *R* point). The electron partial DOS indicated that the H-s and Ca-s states make significant contributions to the valence and conduction bands, respectively. The harmonic phonon dispersions and DOS calculations revealed that the cubic phase is dynamically unstable at 0 K with the negative phonon energies in the whole range of phonon BZ. However, the negative phonon energies were renormalized to be real by the SCP calculations, indicating that the ACaH_3_ can be dynamically stabilized in the cubic phase at elevated temperatures. Based on the DFPT calculations of elastic constants, it was demonstrated that the cubic phase of ACaH_3_ is mechanically stable in accordance with the Born's stability criteria. Finally, we investigated the H_2_ storage capacities and decomposition temperature by considering the quantum effect, finding that *ρ*_g_ (*ρ*_v_) is 5.99 and 4.54 wt% (63.77 and 60.93 g L^−1^), and *T*^q^_des_ is 453.76 and 688.16 K for A = Li and Na, respectively. Based on such calculations, it was concluded that the hydride perovskite LiCaH_3_ is a potential candidate for the onboard hydrogen storage application with the high gravimetric and volumetric capacities of 5.99 wt% and 63.77 g L^−1^ and a suitable dehydrogenation temperature of 453.76 K.

## Data availability

The data supporting this article have been included as part of the ESI.†

## Author contributions

Sol-Hyang Ri and Un-Gi Jong developed the original project, performed the DFT calculations and drafted the first manuscript. Un-Gi Jong and Un-Ryong Rim supervised the work. All authors reviewed the manuscript.

## Conflicts of interest

There are no conflicts to declare.

## Supplementary Material

RA-015-D5RA01810B-s001
