# Supplementary material for: Perovskite-type hydrides ACaH3 (A = Li, Na): computational investigation on materials properties for hydrogen storage applications
Source: RSC Adv. 2025 Jun 6;15(24):19245–53. doi: 10.1039/d5ra01810b (PMC12143255; doi:10.1039/d5ra01810b)
Supplement: RA-015-D5RA01810B-s001 [file RA-015-D5RA01810B-s001.pdf]

Supplementary Information (SI) for RSC Advances.
This journal is © The Royal Society of Chemistry 2025

# Electronic Supplementary Information (ESI) – Perovskite-type hydrides $ACaH_3$ (A=Li, Na): Computational investigation on materials properties for hydrogen storage applications

Sol-Hyang Ri,‡[a] Un-Gi Jong,‡[b*] Thae-Song Im,[c] and Un-Ryong Rim,[d†]

[a] *Faculty of Distance Education, Kim Chaek University of Technology, Pyongyang, PO Box 76, Democratic People's Republic of Korea.*
[b] *Faculty of Materials Science, Computational Materials Design (CMD), Kim Il Sung University, Pyongyang, PO Box 76, Democratic People's Republic of Korea.*
[c] *Faculty of Metal Engineering, Kim Chaek University of Technology, Pyongyang, PO Box 76, Democratic People's Republic of Korea.*
[d] *Institute of Ocean Engineering, Kim Chaek University of Technology, Pyongyang, PO Box 76, Democratic People's Republic of Korea.*

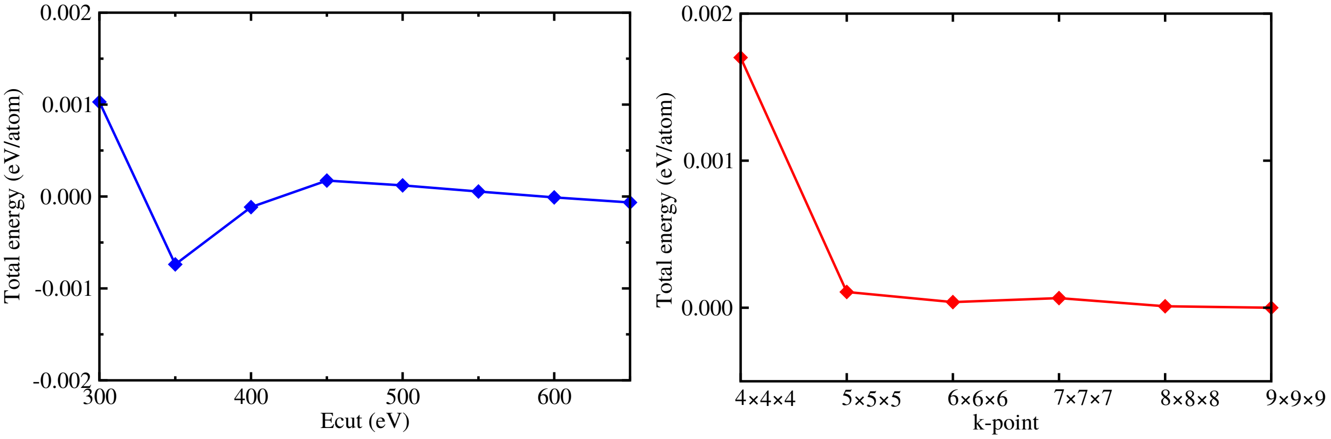


Figure S1 Convergence tests of total energy per atom for a unit cell containing 5 atoms according to the sizes of the energy cutoff and *k*-point mesh for the hydride perovskite $ACaH_3$ (A = Li, Na).

*Un-Gi Jong, Email: ug.jong@ryongnamsan.edu.kp
†Un-Ryong Rim, Email: lur8971@star-co.net.kp ‡ These authors contributed equally.

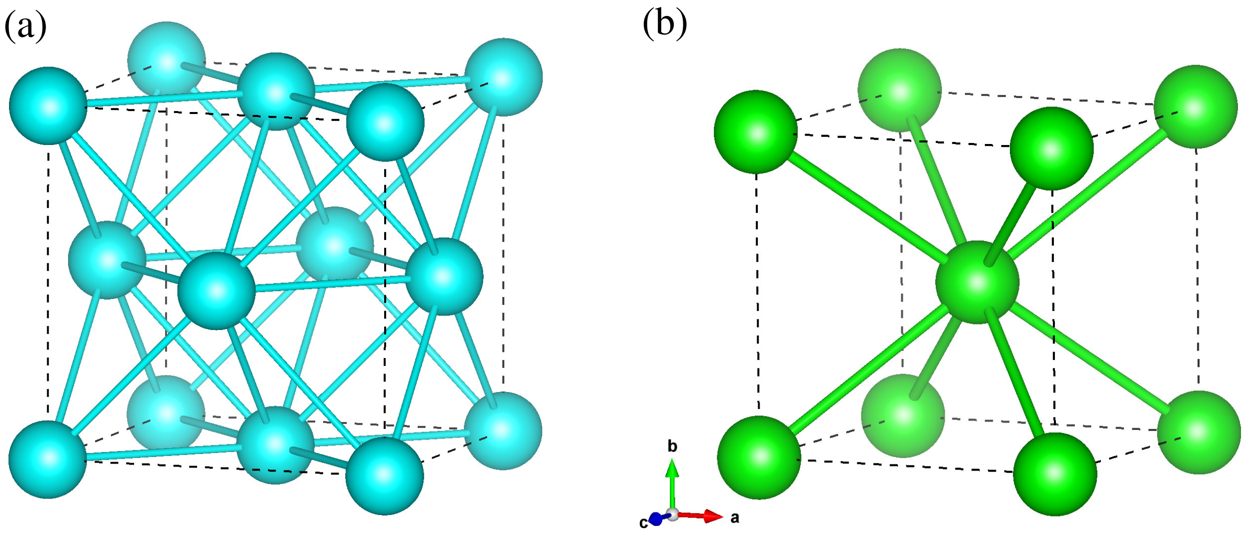


Figure S2 Crystalline structures of (a) the cubic Ca and (b) A compounds with the $Fm\bar{3}m$ and $Im\bar{3}m$ space group, respectively (A = Li, Na).

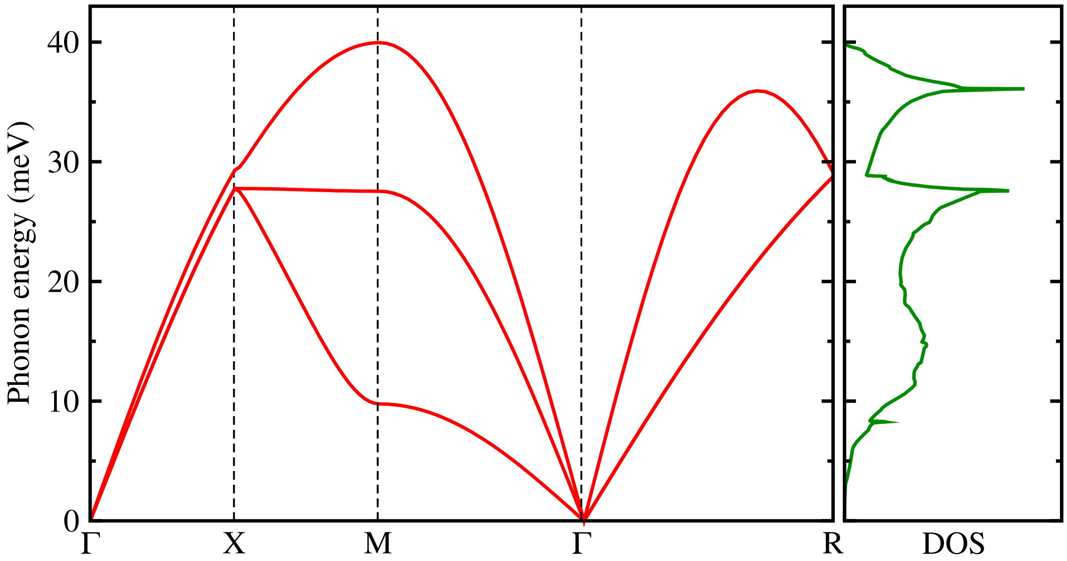


Figure S3 Phonon dispersion curves and phonon density of states (DOS) for the cubic Li with the $Im\bar{3}m$ space group.

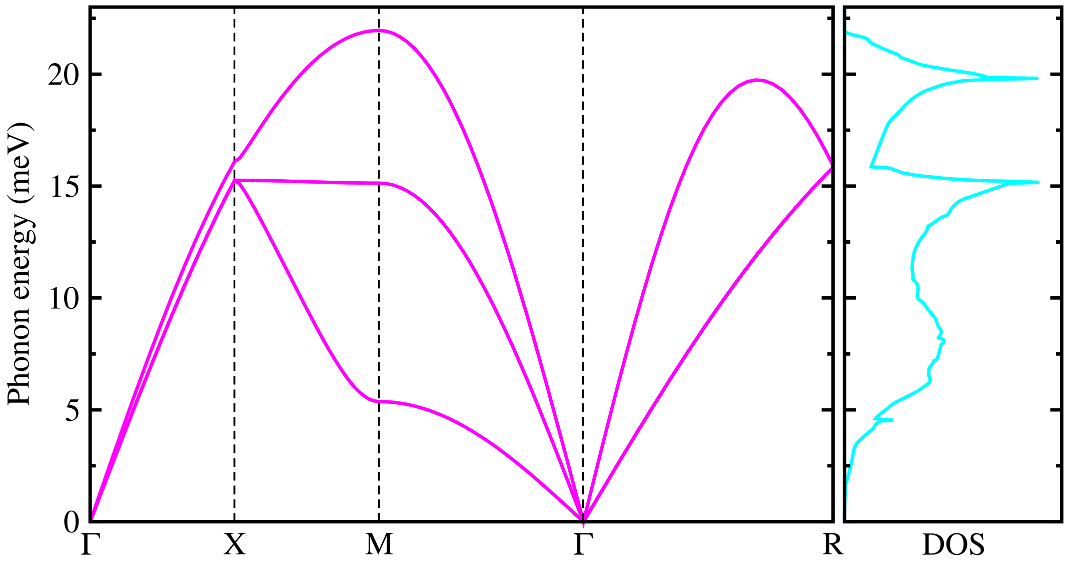


Figure S4 Phonon dispersion curves and phonon density of states (DOS) for the cubic Li with the $Im\bar{3}m$ space group.

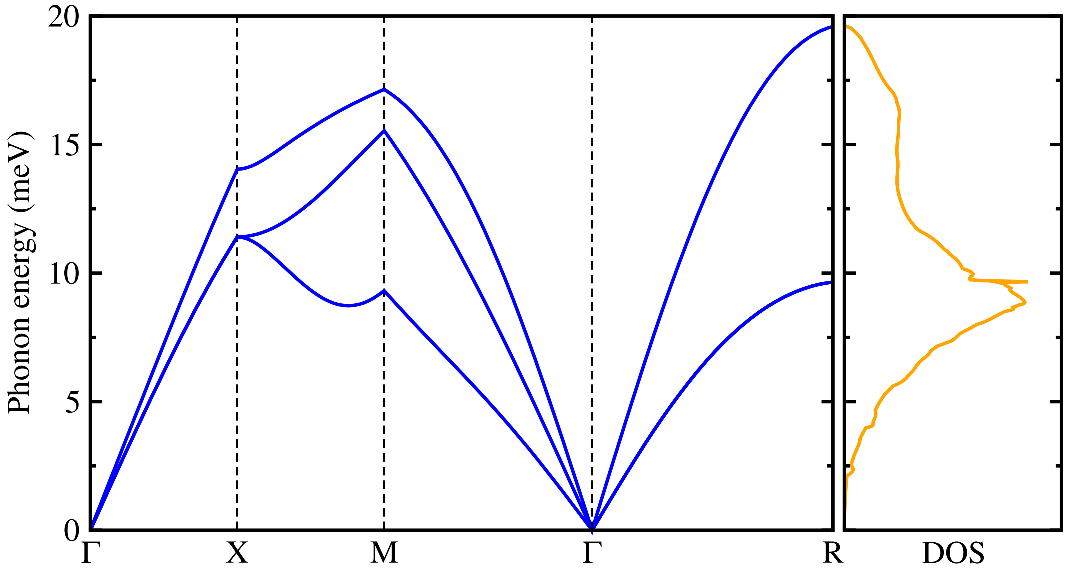


Figure S5 Phonon dispersion curves and phonon density of states (DOS) for the cubic Li with the $Fm\bar{3}m$ space group.

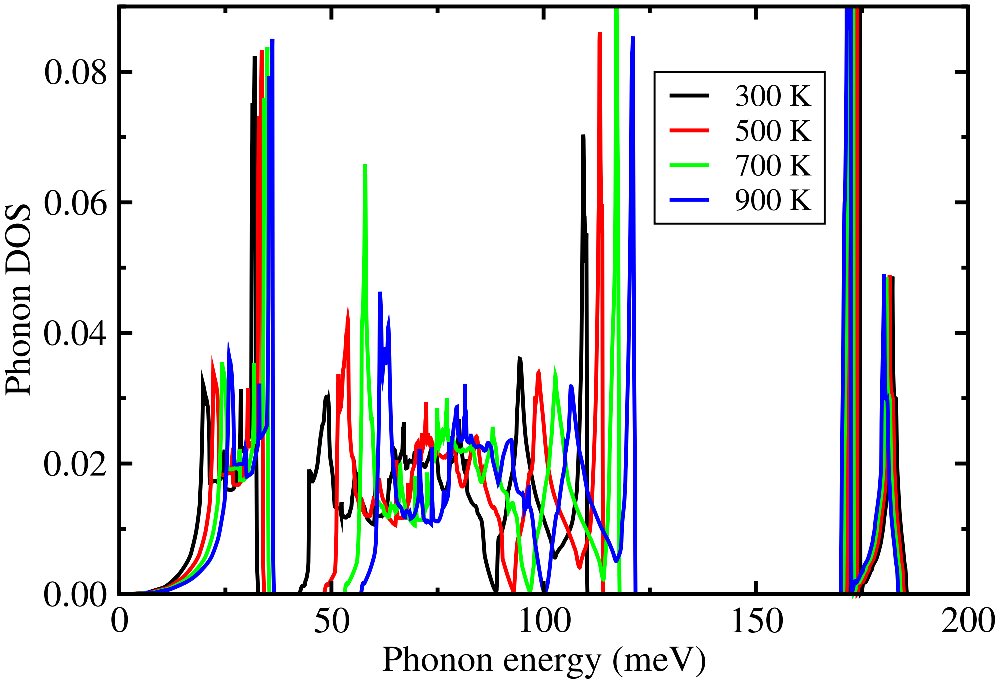


Figure S6 Total phonon density of states (DOS) calculated at 300, 500, 700 and 900 K for the cubic $LiCaH_3$.

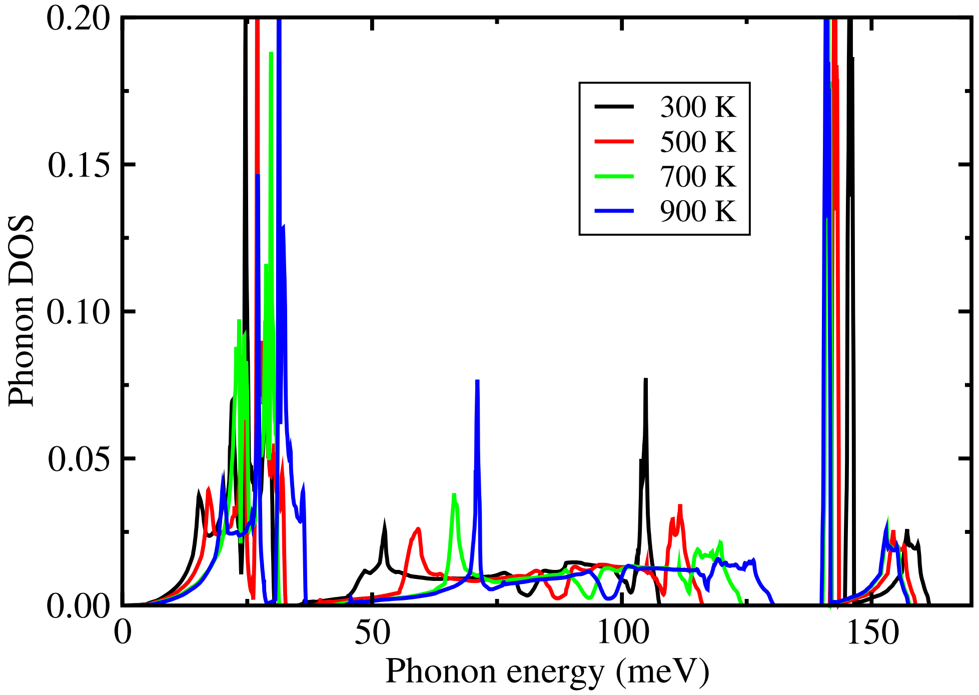


Figure S7 Total phonon density of states (DOS) calculated at 300, 500, 700 and 900 K for the cubic $NaCaH_3$.

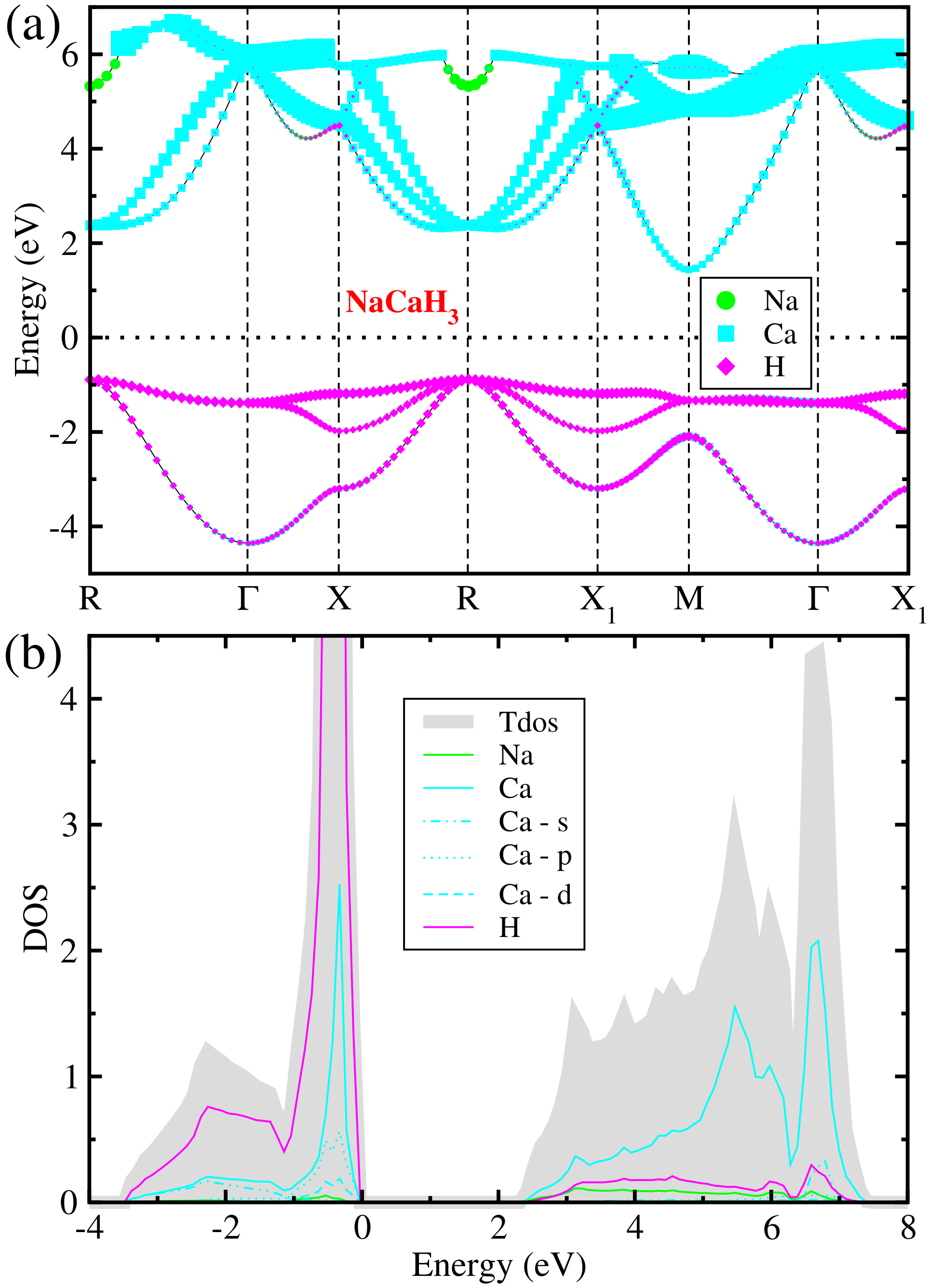


Figure S8 Atom-resolved electronic band structure and (b) partial density of states (DOS) calculated with the PBEsol functional for the hydride perovskite $NaCaH_3$ in the cubic phase.
